# *VcFT-*induced mobile florigenic signals in transgenic and transgrafted blueberries

**DOI:** 10.1038/s41438-019-0188-5

**Published:** 2019-09-11

**Authors:** Guo-qing Song, Aaron Walworth, Tianyi Lin, Qiuxia Chen, Xiumei Han, L. Irina Zaharia, Gan-yuan Zhong

**Affiliations:** 1 Plant Biotechnology Resource and Outreach Center, Department of Horticulture, Michigan State University, East Lansing, MI 48824 USA; 2 Aquatic and Crop Resource Development, National Research Council of Canada, Saskatoon, SK S7N 0W9 Canada; 3 Grape Genetics Research Unit, USDA-ARS, Geneva, NY 14456 USA

**Keywords:** Molecular engineering in plants, Gibberellins

## Abstract

*FLOWERING LOCUS T* (*FT*) can promote early flowering in annual species, but such role has not been well demonstrated in woody species. We produced self and reciprocal grafts involving non-transgenic blueberry (NT) and transgenic blueberry (T) carrying a 35S-driven blueberry *FT* (*VcFT*-OX). We demonstrated that the transgenic *VcFT*-OX rootstock promoted flowering of non-transgenic blueberry scions in the NT (scion):T (rootstock) grafts. We further analyzed RNA-Seq profiles and six groups of phytohormones in both NT:T and NT:NT plants. We observed content changes of several hormone metabolites, in a descending order, in the transgenic NT:T, non-transgenic NT:T, and non-transgenic NT:NT leaves. By comparing differential expression transcripts (DETs) of these tissues in relative to their control, we found that the non-transgenic NT:T leaves had many DETs shared with the transgenic NT:T leaves, but very few with the transgenic NT:T roots. Interestingly, a number of these shared DETs belong to hormone pathway genes, concurring with the content changes of hormone metabolites in both transgenic and non-transgenic leaves of the NT:T plants. These results suggest that phytohormones induced by *VcFT*-OX in the transgenic leaves might serve as part of the signals that resulted in early flowering in both transgenic plants and the non-transgenic NT:T scions.

## Introduction

Florigen was originally defined to be graft-transmissible hormones or hormone-like molecules involved in long-distance regulation of flowering^1–3^. Initially, florigen was hypothesized to be a specific ratio of known hormones and metabolites despite a lack of convincing molecular evidence^1–3^. With the discovery of *FLOWERING LOCUS T* (*FT*) in *Arabidopsis*^4,5^, and functional analysis of *FT* or its orthologues in many plant species^6–8^, and subsequent demonstration of *FT* as a promoter of flowering with short-distance mobility from leaves to adjacent meristems^9–11^, *FT* emerged as a top candidate for florigen.

However, accepting “FT-as-florigen” is still controversial. The main contentious issue is the mechanism by which FT signals are transmitted from source leaf to recipient meristems to promote flowering. For herbaceous plants, many reports have demonstrated that FT proteins, instead of *FT* RNAs, acted as the mobile florigenic signals mainly by short-distance transport (e.g., from leaves to their adjacent buds)^9,12–19^. Two other reports, however, suggested that both FT proteins and *FT* RNAs could be transmitted^20,21^. For woody plants, the evidence for FT-based flowering promotion and FT proteins or *FT* RNAs as florigenic signals is not consistent. For example, in woody shrubs, while overexpression of the *Jatropha FT* in transgenic *Jatropha* rootstock promoted flowering in recipient scions^22^, such a phenomenon was not observed in recipient scions grafted on transgenic cassava (*Manihot esculenta* Crantz) containing a constitutively expressed *Arabidopsis FT*^23^. In both reports, potential transport of *Jatropha* FT proteins or *FT* RNAs as florigenic signals were not analyzed^22,23^. Various attempts to promote flowering in recipient scions by expressing *FT* or *FT* orthologues from poplar (*Populus trichocarpa PpFT*) in transgenic rootstocks of poplar^24^, apple^25^, and plum^26^ have been unsuccessful. Among these studies, *PpFT* mRNAs from transgenic rootstocks were only detected in non-transgenic scions of micro-grafted apple plants^25^. Very disappointedly, no further studies on long-distance transport of FT proteins or *FT* mRNAs in transgrafted woody plants have been reported. Equally disappointed is that no satisfactory explanations are available for why overexpression of transgenic *FT* in rootstocks could not promote flowering of non-transgenic scions in woody species^9^.

While florigen was initially believed to be hormones or hormone-like molecules, surprisingly, little connection has been made between them. It is well known that phytohormones [e.g., abscisic acid (ABA), auxin, cytokinin, ethylene, and gibberellins (GAs)] play important roles in regulating plant flowering and stature formation^27^. For example, mutations resulting in reduced GA biosynthesis or increased GA degradation often produce dwarf plants with delayed flowering^28–32^. Other phytohormone genes [e.g., auxin^33,34^, cytokinin^3,35,36^, ethylene^37^, brassinosteroid^38,39^, jasmonic acid (JA)^40^, nitric oxide^41^, peptide hormone^42^, and salicylic acid (SA)^43,44^] also affect plant flowering and size. Because of small molecular sizes and diverse physiological properties, phytohormones are known to be an important class of biochemical signals in plant development and growth

To understand FT-based flowering promotion in woody species, we previously generated transgenic blueberry with overexpressed *VcFT* (*VcFT*-OX) and showed *VcFT*-induced flowering in transgenic blueberry^45^. In this study, our objective was to investigate whether *VcFT*-OX plants can be used as rootstocks to induce flowering in non-transgenic scions and, if yes, what would likely be the florigenic signals moving from rootstocks to scions to promote flowering. Through grafting, we demonstrated the ability of the transgenic *VcFT* blueberry, when it was uses as a rootstock, to induce flowering of non-transgenic blueberry scions. We further revealed that transgenic *VcFT* was highly expressed in transgenic leaves, but surprisingly suppressed in the transgenic roots. Moreover, we identified several hormone genes, among others, differentially expressed and the expression levels of some of these genes corelated with the content changes of corresponding hormones between the transgenic rootstock and non-transgenic scion. We concluded that phytohormones were likely involved in *FT*-mediated flowering and could be a part of the florigenic signals moving from leaves to adjacent buds or apical meristems for inducing flowering in blueberry.

## Results

### *VcFT-*OX promoted flowering in transgrafted scions

**Fig. 1:**
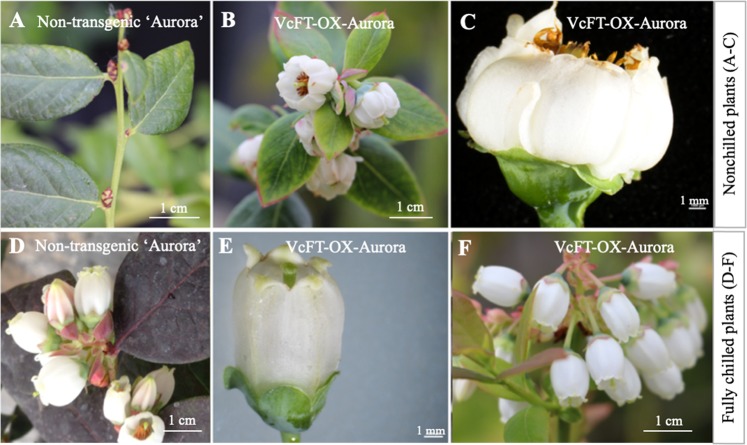
Flowers of 6-year-old, non-transgenic ‘Aurora’ and 3-year-old VcFT-OX-Aurora plants. **a** Nonchilled ‘Aurora’ flower buds. **b**, **c** Flowers of nonchilled VcFT-OX-Aurora. **d** Flowers of fully chilled non-transgenic ‘Aurora’. **e**, **f** Flowers of fully chilled VcFT-OX-Aurora. Note: few flower buds appeared in the 3-year-old VcFT-OX ‘Aurora’ plants

We previously demonstrated that overexpression of *VcFT*-induced precocious flowering and dwarfing in all transgenic blueberry ‘Aurora’ plants^46^. We subsequently analyzed the gene expression patterns of a representative transgenic event (hereafter noted as *VcFT*-OX-Aurora) and revealed numerous differentially expressed genes (DEGs) associated with flowering and phytohormone pathways^45,47^. Due to the well-defined phenotypic changes and the availability of the micropropagated plants, in this study, 3-year-old plants of this representative transgenic *VcFT*-OX-Aurora (T) and non-transgenic ‘Aurora’ (NT) were self [i.e., T (scion):T (rootstock) and NT:NT] and reciprocally (i.e., NT:T and T:NT) grafted. Ungrafted 6-year-old NT plants with flower buds, as expected, did not flower without fulfilling chilling requirement (Fig. 1a), while ungrafted, nonchilled T plants showed flowering, although some abnormal flowers with wrinkled petals were observed (Fig. 1b, c). The fully chilled T plants, however, had well-formed flowers and flowered normally (Fig. 1e, f), compared with the 6-year-old NT plants (Fig. 1d). The fully chilled 3-year-old ungrafted and NT:NT plants did not flower due to the lack of flower buds.

**Fig. 2:**
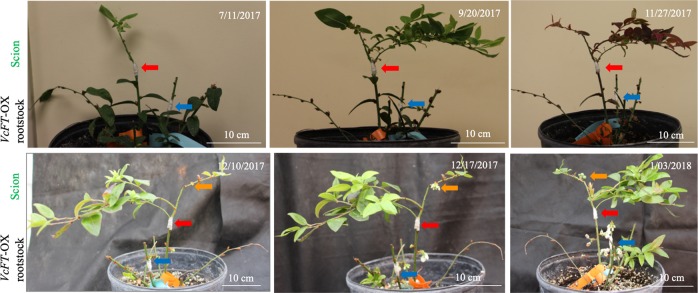
A VcFT-OX-Aurora plant with the graft combinations of T:T and NT:T. The shown T:T and NT:T grafting was made on May 6, 2017; after receiving chilling treatment from September 20 to November 27, 2017, flower buds on the non-transgenic NT scion were observed to break on December 10, 2017. Red, blue, and orange arrows show the grafting unions of NT:T, T:T and the flowers or fruits on the NT scion, respectively. No flowers were observed in non-transgenic ‘Aurora’ NT or NT:NT plants during the whole observation period from November 27, 2017 to May 23, 2018 (Fig. S1)

Grafting is not a common practice for blueberry bushes, but it has potential to increase abiotic stress tolerance for cultivated blueberry cultivars^48^. In this study, the survival rate of grafted scions on T rootstocks ranged from 16.7 to 60.0% 2 weeks after grafting. In contrast, all T:NT grafting failed. The survived transgenic scions on T:T plants were similar to the ungrafted T plants, producing no new shoots due to the lack of leaf buds. In contrast, the survived non-transgenic scions on both NT:T and NT:NT plants formed new shoots after four and half months (Fig. 2). Under non-chilling conditions, none of the NT shoots on T rootstocks produced flowers. However, after receiving full chilling (~1600 chilling hours) for 2 months and transferring to a warm greenhouse, flowering was observed from buds on the NT shoots of all the six NT:T plants within 2 weeks. In contrast, no flowers were observed on the NT scions (grafted or non-grafted) on any NT:NT plants or on ungrafted ‘Aurora’ plants (Fig. 2, Fig. S1). Apparently, the NT:T plants were able to promote flowering of non-transgenic scions when exposed to chilling. These results suggested that the *VcFT*-OX in the transgenic rootstock of the NT:T plants initiated florigenic signals from either transgenic leaves or transgenic roots that went through a long-distance transportation for hastening flower bud formation and flowering in the non-transgenic scions.

### *VcFT-*OX altered the contents of some major phytohormones

Florigen was initially believed to be hormones or hormone-like molecules^2^. In order to determine potential roles of phytohormones in promoting flowering, 41 metabolites of six phytohormone groups [ABA (7 metabolites), auxin (6), cytokinin (10), GA (14), JA (3), and SA (1)] were quantified in both transgenic and non-transgenic tissues of the NT:T plants as well as in the control plants NT:NT.

**Fig. 3:**
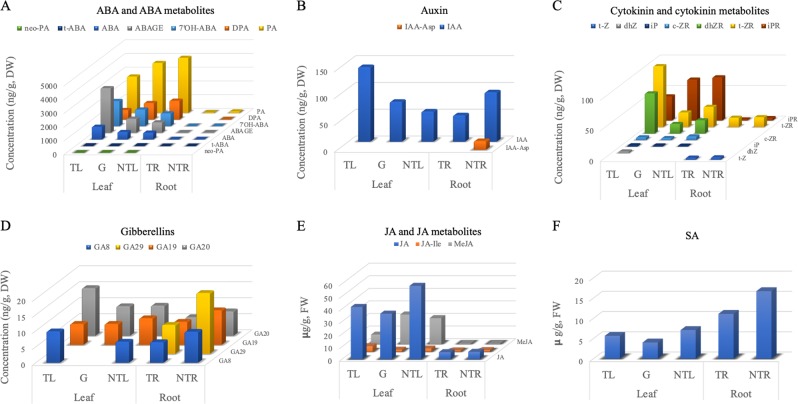
Content variation of the hormones detected in various tissues of NT:T and NT:NT plants. **a** ABA and six ABA metabolites (ABAGE: Abscisic acid glucose ester, DPA: Dihydrophaseic acid, PA: Phaseic acid, 7′OH-ABA: 7′-Hydroxy-abscisic acid, neo-PA: *neo*-Phaseic acid, and *t*-ABA: *trans*-Abscisic acid). **b** Six auxin and metabolites [IAA: Indole-3-acetic acid, IAA-Asp: N-(Indole-3-yl-acetyl)-aspartic acid, IAA-Glu: N-(Indole-3-yl-acetyl)-glutamic acid, IAA-Ala: N-(Indole-3-yl-acetyl)-alanine, IAA-Leu: N-(Indole-3-yl-acetyl)-leucine, and IBA: Indole-3-butyric acid] were measured, but only IAA and IAA-Asp were detected and shown. **c** 10 cytokinin and cytokinin metabolites [*t*-ZOG: (t*rans*) Zeatin-O-glucoside, *c*-ZOG: (*cis*) Zeatin-O-glucoside, *t*-Z: (*trans*) Zeatin, *c*-Z: (*cis*) Zeatin; dhZ: Dihydrozeatin, *t*-ZR: (*trans*) Zeatin riboside, *c*-ZR: (*cis*) Zeatin riboside, dhZR: Dihydrozeatin riboside, iP: Isopentenyladenine, and iPR: Isopentenyladenosine] were measured, six of them (*t*-Z, dhZ, *t*-ZR, *c*-ZR, dhZR, iP, and iPR) were detected and shown. **d** Fourteen gibberellins (GA1, GA3, GA4, GA7, GA8, GA9, GA19, GA20, GA24, GA29, GA34, GA44, GA51, and GA53) were measured, but only GA8, GA19, GA20, and GA29 were detected and shown. **e** JA and 2 JA metabolites (MeJA: Methyl jasmonate, JA-Ile: jasmonoyl isoleucine were measured and shown. **f** Salicylic acid. TL: transgenic leaves from NT:T grafts. G: non-transgenic leaves from NT:T grafts. NTL: non-transgenic NT:NT leaves. TR: transgenic NT:T roots. NTR: non-transgenic NT:NT roots. DW: dry weight. FW: fresh weight

We analyzed seven ABA and ABA metabolites and they were all detected in both transgenic and non-transgenic leaves of NT:T plants (note that transgenic leaves were from the transgenic rootstocks T and non-transgenic leaves from the grafted non-transgenic scions NT) as well as in the non-transgenic NT:NT leaves. The total accumulation level of ABA and its metabolites in the transgenic leaves was higher than those in the non-transgenic leaves from the NT:T plants, but the trend varied among individual metabolites (Fig. 3a), with dihydrophaseic acid (DPA) and phaseic acid (PA) showing lower accumulation in the transgenic leaves. The reduction of DPA in the transgenic leaves was statistically significant (*p* *=* 0.01) (Fig. 3, Table 1). Overall accumulation of the seven ABA metabolites was much lower in the roots than in the leaves. All seven metabolites were present in NT:NT roots, and six of them, except for DPA, were detected in the transgenic NT:T roots (Fig. 3, Table 1). ABA showed a significant decrease (*p* = 0.02) in the transgenic NT:T roots and the total level of ABA and its metabolites in the transgenic roots was about half of that observed in the non-transgenic roots (Fig. 3, Table 1). All seven ABA and ABA metabolites were also detected in the transgenic NT:T leaves, although none of them had significant accumulation changes compared with the non-transgenic leaves of both NT:T and NT:NT plants (*p* = 0.05) (Table 1). However, the accumulation levels of the seven metabolites were mostly between transgenic and non-transgenic leaves of the NT/T, with three being increased and two decreased (Fig. 3a).

**Table 1:** Statistical analysis of the content variation of the hormones detected in various tissues of NT:T and NT:NT plants

		ng/g dry weight				
Hormone group	Hormones and metabolites	Transgenic NT:T leaves	Non-transgenic NT:T leaves	Non-transgenic NT:NT leaves	Transgenic NT:T roots	Non-transgenic NT:NT roots
ABA	ABA	927.3 ± 451.8 a*	544.7 ± 174.5 a	499.7 ± 88.2 a	18.7 ± 1.2 c	23.7 ± 2.1 b
	DPA	697.3 ± 91.6 b	1204.0 ± 110.6 a	1371.0 ± 262.1 a	nd	7.7 ± 13.3 c
	ABAGE	3252.0 ± 1862.9 a	1023.0 ± 110.0 a	763.3 ± 188.9 a	25.3 ± 4.7 b	37.7 ± 8.3 b
	PA	2645.7 ± 618.2 a	3635.7 ± 535.9 a	4010.0 ± 1030.1 a	43.7 ± 21.1 b	110.3 b
	7’OH-ABA	1833.0 ± 1561.2 a	1213.0 ± 418.8 a	934.3 ± 96.1 a	8.9 ± 4.5 b	19.3 ± 9.3 b
	neo-PA	8.0 ± 4.4 a	6.7 ± 4.9 a	4.7 ± 0.8 a	nd	nd
	t-ABA	50.3 ± 22.0 a	25.3 ± 18.3 a	54.3 ± 20.6 a	5.3 ± 0.6 b	4.7 ± 0.6 b
Cytokinin	t-ZR	99.7 ± 23.2 a	24.0 ± 8.9 b	33.0 ± 18.1 b	15.3 ± 2.5 c	16.0 ± 6.6 c
	c-ZR	4.7 ± 0.6 b	3.3 ± 1.5 b	6.3 ± 1.1 a	nd	nd
	dhZR	65.7 ± 15.3 a	15.7 ± 5.9 b	21.3 ± 8.1 b	nd	nd
	iP	1.7 ± 0.6 a	1.7 ± 0.6 a	1.3 ± 0.6 a	nd	nd
	iPR	39.7 ± 2.1 a	67.0 ± 15.6 a	70.7 ± 33.3 a	1.7 ± 0.6 b	4.0 ± 1.0 c
	t-Z	nd	nd	nd	3.3 ± 1.2 a	4.0 ± 1.0 a
	dhZ	1.0 ± 1.7 a	nd	nd	nd	nd
Auxin	IAA	139.0 ± 41.6 a	74.7 ± 28.4 ab	56.3 ± 35.9 ab	49.0 ± 41.2 b	92.3 ± 44.1 ab
	IAA-Asp	nd	nd	nd	nd	16.3 ± 10.1 b
GA	GA20	15.0 ± 6.9 a	9.3 ± 4.2 a	9.7 ± 7.4 a	6.0 ± 1.0 a	6.3 ± 4.0 a
	GA29	nd	nd	nd	3.0 ± 5.2 a	9.7 ± 8.2 a
	GA8	6.7 ± 7.0 a	1.3 ± 2.3 a	3.9 ± 3.6 a	5.7 ± 2.1 a	6.1 ± 3.4 a
	GA19	5.6 ± 1.8 a, b	3.5 ± 3.3 b	6.7 ± 2.5 a, b	7.3 ± 2.1 a, b	10.7 ± 1.2 a
		µg/g fresh weight				
SA	SA	5.7 ± 1.6 a	4.0 ± 1.8 a	7.1 ± 3.7 a	11.1 ± 5.2 a, b	16.7 ± 4.5 b, c
JA	JA	41.5 ± 15.1 a, b	36.2 ± 16.3 a, b	58.3 ± 18.2 a	5.6 ± 2.5 c	5.8 ± 1.9 c
	JA-Ile	4.2 ± 2.1 a	1.5 ± 0.5 a	2.4 ± 1.1 a	0.9 ± 1.3 a	1.2 ± 0.2 a
	MeJA	7.4 ± 3.6 b	23.4 ± 2.9 a	20.5 ± 4.5 a	0.4 ± 0.2 c	0.3 ± 0.1 c

*Nd* non-detectable

*Numbers (means of three biological replicates ± standard deviation) in each row with different letters are significantly different at *p* = 0.05

We assayed six auxins and only indole-3-acetic acid (IAA) and N-(indole-3-yl-acetyl)-aspartic acid (IAA-Asp) were detected (Fig. 3b, Table 1). Biologically active IAA was found in all of the leaf and root samples while IAA-Asp was detected only in the non-transgenic roots. In the NT:T plants, IAA accumulation was the highest in transgenic leaves, followed by non-transgenic leaves and transgenic roots. In the NT:NT plants, roots had higher IAA content than leaves. When NT:T plants were compared with NT:NT plants, non-transgenic NT:T leaves had higher IAA content than the NT:NT leaves (*p* = 0.06). However, IAA content in the transgenic roots showed no significant difference (*p* = 0.28) from the non-transgenic root samples, despite the apparently higher IAA content in the non-transgenic roots (92.3 ng/g DW in the non-transgenic roots versus 49.0 ng/g DW in the transgenic roots) (Table 1). This was likely due to the small sample size in the analysis. The IAA accumulation in the non-transgenic NT:T leaves was intermediate between that of the transgenic NT:T leaves and non-transgenic NT:NT leaves.

Cytokinins were the top candidate for florigen before the discovery of *FT*^3,4^. Seven cytokinins were measured in this study. Six of them were detected in leaves and three in roots (Fig. 3c, Table 1). Of the three cytokinins detected in roots, isopentenyladenosine content showed a significant decrease in the transgenic roots compared with the non-transgenic roots. Of the six cytokinins detected in leaves, all were detected in the transgenic leaves and five in the non-transgenic leaves of both NT:T and NT:NT plants. The content of dihydrozeatin riboside (dhZR) and (*trans*) zeatin riboside (t-ZR) was significantly higher in the transgenic NT:T leaves than in the non-transgenic leaves of either NT:T and NT:NT plants (Table 1).

Gibberellins play significant roles in regulating plant flowering^49,50^. The content of 14 GAs were measured, but only 3 and 4 were detected in the leaf and root samples, respectively (Fig. 3d, Table 1). No significant changes of GAs were found either between transgenic leaves and non-transgenic leaves or between transgenic roots and non-transgenic roots. Compared with non-transgenic NT:NT leaves, transgenic leaves appeared to have less GA19, but more GA8 and GA20, and transgenic roots had less GA19, GA20, and GA29 than non-transgenic roots. In the non-transgenic NT/T leaves, GA19 and GA20, but not GA8, were detected (Table 1).

The hormones of JA and two JA metabolites (Fig. 3e) and SA (salicylic acid, Fig. 3f) were detected in both leaf and root samples (Table 1). Relative to the non-transgenic NT:NT leaves, MeJA showed a significant decrease in transgenic leaves while SA was significantly decreased in transgenic roots. Notably, none of the four compounds measured showed a significant difference between transgenic and non-transgenic leaves of the NT:T plants.

### *VcFT-*OX induced genome-wide differential gene expression

**Fig. 4:**
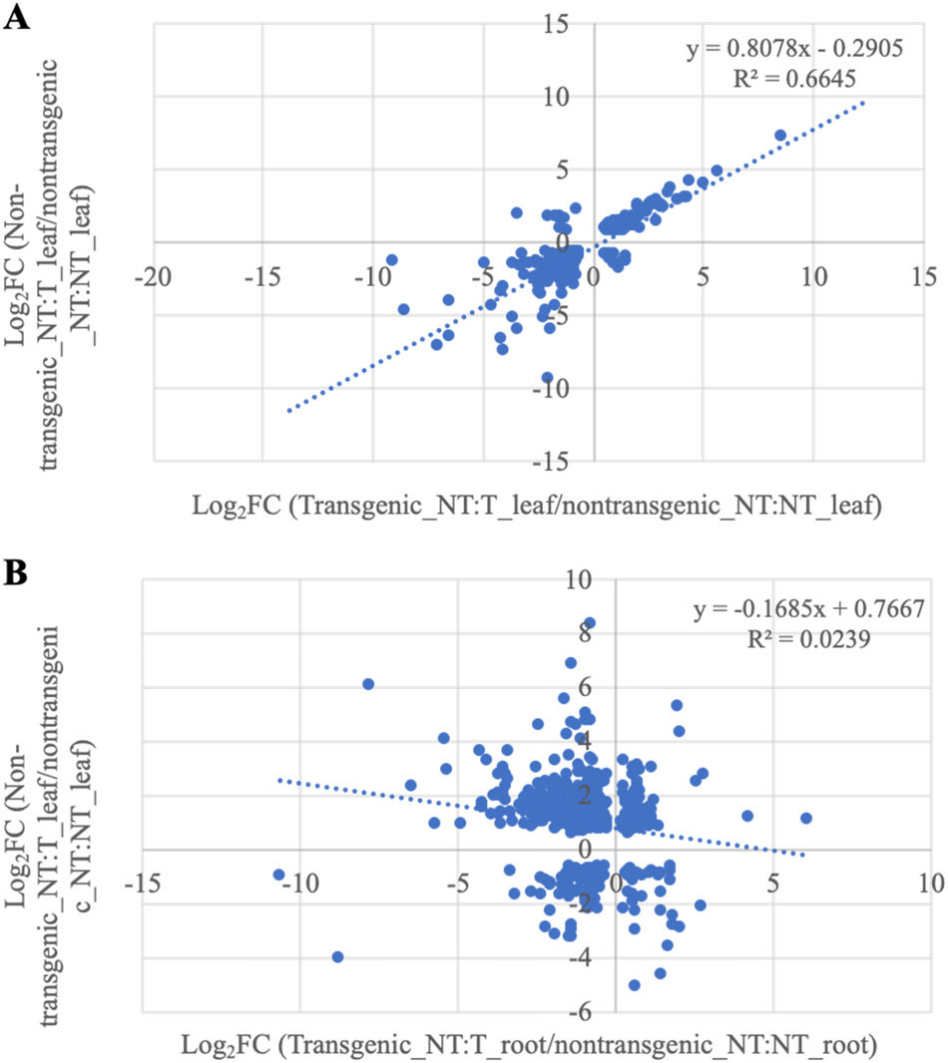
Correlation plots of the fold of changes (Log2) of DETs identified from different tissues of the NT:T grafts. **a** DETs identified in the transgenic leaves of NT:T grafts (*x*-axis) vs. corresponding DETs identified in the non-transgenic leaves of NT:T grafts (*y*-axis). **b** DETs identified in the transgenic roots of NT:T grafts (*x*-axis) vs. corresponding DETs identified in the non-transgenic roots of NT:T grafts (*y*-axis)

To examine *VcFT*-OX induced transcriptomic changes, RNA-Seq profiles of non-transgenic NT:NT leaves, transgenic and non-transgenic NT:T leaves were compared (Fig. S2, Table S1). The comparison between non-transgenic NT:NT leaves and transgenic NT:T leaves resulted in 2421 DE transcripts (DETs) (948 DEGs: hereafter the DEGs refer to the differentially expressed unique genes defined by the Trinotate annotation) (FDR < 0.05), of which *VcFT* was about 5000-fold (FPKM = 107.70) greater in transgenic NT:T leaves compared with non-transgenic NT:NT leaves (FPKM = 0.02) and 962 DETs (420 DEGs) were upregulated in the transgenic leaves (Table S1). In comparison, 1165 DETs (723 DEGs) were found between non-transgenic NT:NT leaves and non-transgenic NT:T leaves, of which only 318 DETs (162 DEGs) showed increased expression in the non-transgenic NT:T leaves (Table S1). A total of 299 DETs (150 DEGs) were overlapped between the two comparisons, and the expression changes (Log_2_FC values) of these 299 DETs exhibited a strong positive correlation (*y* = 0.8078 × −0.2905, *R* = 0.82, *p*-value < 4.4e−4) between the two groups (Fig. 4a, Table S2).

In a previous study, we observed in the blueberry cultivar ‘Legacy’ that *VcFT* in nonchilled flower buds had higher expression (FPKM = 34.9) than young leaves (FPKM: 0.2) and flowers (FPKM: 0.9)^47^. Similarly, in this study, non-transgenic NT:NT plants had low *VcFT* expressions in leaves (FPKM: 0.02) and roots (FPKM: 0.08). The *VcFT* expression in the transgenic leaves and roots of NT:T plants were 107.70 and 0.12 FPKM, respectively. The accumulation of *VcFT* RNA messages in the transgenic roots was surprisingly low, but in line with the general observation that *FT* expression is much suppressed in roots^51^. Transcriptome comparison between the transgenic and non-transgenic roots resulted in 18,275 DETs of 5516 genes (Table S1). Since *VcFT* expression were very low in both transgenic and non-transgenic roots, such many DETs observed between their comparisons (Table S1) was very intriguing. A common set of 517 DETs of 274 genes were identified in the comparison between the 723 DEGs in non-transgenic NT:T leaves and 18,275 DETs in transgenic NT:T roots (Fig. S2). The expression changes (Log_2_FC values) of these 517 DETs showed a very weak positive linear correlation (*y* = −0.1685 × +0.7667, *R* = 0.15, *p*-value < 2.2e−16) between two groups of the DETs (Fig. 4b), suggesting that the changes of the 517 DETs in non-transgenic NT:T leaves were not much related to the DETs from transgenic roots. A gene network analysis of the 299 DETs shared in the comparisons of non-transgenic NT:NT leaves versus transgenic and non-transgenic NT:T leaves, respectively, revealed 91 gene ontology (GO) terms. Among them, 18 were related to flower development and 7 related to hormones (Fig. S3, Tables S2, S3), indicating that hormone pathway genes were significantly involved in the *VcFT*-triggered flowering processes.

In the differential expression analysis, we applied a very restrictive FDR value to declare significances for various comparisons. We found that many transcripts which were not expressed in one of the paired tissues in the comparisons often did not pass the cut-off threshold. However, these transcripts may represent some genes which were uniquely suppressed or enhanced in the tissue because of the introduction of the *VcFT*. We examined these transcripts and found 760 which were expressed in the non-transgenic NT:NT leaves (all three biological replicates with FPKM > 0.1) but suppressed in the transgenic leaves (all three replicates with FPKM = 0) (Table S4). In contrast, 216 were not detected in the non-transgenic NT:NT leaves but expressed in the transgenic leaves. Similarly, 193 transcripts were expressed in the non-transgenic NT:T leaves, but suppressed in the non-transgenic NT:NT leaves while expression behavior of 14 other transcripts were reversed in the two tissues. Interestingly, 187 (177 suppressed and 10 enhanced) of the 207 transcripts of the non-transgenic NT:T leaves (193 suppressed and 14 enhanced) were shared with the 876 uniquely suppressed or enhanced transcripts in the transgenic leaves (760 suppressed and 216 enhanced). In other words, about 90% of the uniquely suppressed or enhanced transcripts in the non-transgenic NT:T leaves were found in the transgenic leaves, which accounted for about 21% of the total uniquely suppressed or enhanced transcripts in the transgenic leaves. On the other hand, only 25 transcripts (<3%) were shared between the transgenic leaves (876) and transgenic roots of the NT:T plants (1480). To our surprise, there were no other overlaps of such uniquely suppressed or enhanced transcripts between non-transgenic leaves and transgenic roots of the NT:T plants, except for two (suppressed) which were shared by all three tissues. These results strongly suggested that DETs in the NT:NT leaves were much more related to those in the transgenic leaves and had little connection with those in the transgenic roots. Since many of these shared transcripts could not be annotated with the current known databases, their functions and significance in promoting flowering are unknown.

### Differential expression of flowering, hormones, and sugar pathway genes

By using flowering pathway genes of *Arabidopsis* as inquiries^47^, we found 41 DETs in the differential expression analysis of transgenic leaves vs. non-transgenic NT:NT leaves, 10 DETs in the non-transgenic NT:T leaves vs. non-transgenic NT:NT leaves, and 262 DETs in the transgenic roots vs. non-transgenic roots (Table S5). Among the 10 DETs in the NT:T leaves, 5 DETs (4 DEGs) were shared with the transgenic leaves (Table 2). One of them is *SOC1* which is a well-known target of *FT* in flowering^52^. Interestingly, all four genes showed much reduced expression in both transgenic and non-transgenic leaves of the NT:T plants, compared with the non-transgenic NT:NT leaves. On the other hand, there was only one DEG, AT5G13480.2, shared between the non-transgenic leaves and transgenic roots in the NT:T plants. AT5G13480.2 is a known regulator in flowering^53^. There were eight flowering-related DETs shared between the transgenic leaves and transgenic roots. Similarly, we found 20 DETs of sugar pathway genes in the comparison of transgenic NT:T leaves vs. non-transgenic NT:NT leaves, 13 DETs in the non-transgenic NT:T leaves vs. non-transgenic NT:NT leaves, and 164 DETs in the transgenic roots vs. non-transgenic roots (Table S6). Most of these DETs were downregulated in the transgenic tissues. Surprisingly, there was no overlapped sugar DETs between the transgenic and non-transgenic leaves in the NT:T plants, while 6 and 10 were shared between the non-transgenic leaves and transgenic roots and between the transgenic leaves and transgenic roots in the NT:T plants, respectively. In contrast to the flowering and sugar genes, by using the reference of the 245 biosynthetic pathway genes representing nine groups of phytohormones (i.e., ABA, auxin, brassinosteroid, cytokinin, gibberellin, ethylene, JA, SA, and strigolactones) of *A. thaliana* (http://hormones.psc.riken.jp), we found 98, 71, and 463 DETs of hormone pathway genes in the transgenic leaves, non-transgenic leaves, and transgenic roots of the NT:T plants, respectively (Table 3). There were 23 DETs of hormone genes shared between non-transgenic and transgenic leaves, 34 between non-transgenic leaves and transgenic roots, and 51 between transgenic leaves and transgenic roots in the NT:T plants. Interestingly, four DETs were shared among all three tissues, involving one ABA, one cytokinin, and two GA genes (Table 3).

**Table 2:** Differentially expressed transcripts of flowering pathway genes detected in the non-transgenic leaves of NT:T vs. NT:NT plants

Query_id	Subject_id	*E*-value	Log_2_FC	*p*-value	FDR	Annotation by Trinotate	Gene_name
AT5G13480.2	c88585_g1_i1	3.00E−20	−1.07	2.68E−07	0.00013	COB23_ARATH	FY
AT5G24470.1	c92704_g6_i2	2.00E−116	−2.09	3.56E−10	0.00000	PRR95_ORYSJ	APRR5, PRR5
AT5G24470.1	c92704_g6_i1	1.00E−116	−2.23	1.05E−08	0.00001	APRR5_ARATH	APRR5, PRR5
AT4G22950.1	c94107_g4_i1	7.00E−33	−2.35	1.38E−07	0.00007	SOC1_ARATH	AGL19, GL19
AT4G34000.1	c89508_g1_i2	3.00E−50	−1.68	6.37E−08	0.00004	AI5L5_ARATH	ABF3, DPBF5
AT2G45660.1	c94107_g3_i3	1.00E−23	−6.54	6.13E−05	0.01222	–	AGL20, SOC1
AT2G23080.1	c98469_g3_i1	7.00E−22	−0.85	4.17E−04	0.05358	Y1461_ARATH	AT2G23080.1
AT1G13260.1	c83982_g1_i2	6.00E−130	−1.24	2.64E−06	0.00095	RAV1_ARATH	RAV1, EDF4
AT2G45660.1	c88293_g4_i5	3.00E−34	−2.84	1.02E−06	0.00041	–	AGL20, SOC1
AT4G22950.1	c94107_g4_i2	1.00E−20	−4.64	8.67E−06	0.00255	SOC1_ARATH	AGL19, GL19

*Log*_*2*_*FC* Log_2_ fold change (NT:T/NT:NT), *CPM* count per million reads, *FDR* false discovery rate

**Table 3:** Differentially expressed transcripts of phytohormone genes detected in the comparisons of transgenic leaves from NT:T vs. non-transgenic leaves from NT:NT, non-transgenic leaves from NT:T vs. non-transgenic leaves from NT:NT, and transgenic roots from NT:T vs. non-transgenic roots form NT:NT

Query id	Subject id	Log_2_ (NT:T transgenic leaves/NT:NT leaves)	Log_2_(NT:T non- transgenic leaves/NT:NT leaves)	Log_2_(NT:T root/ NT:NT root)	Annotation by Trinotate	Hormone	Gene_name
AT2G29090.1	c80869_g4_i1	#N/A	−1.79	−0.68	ABAH1_ARATH	ABA	CYP707A2
AT2G29090.1	c84314_g2_i1	#N/A	−1.63	#N/A	ABAH1_ARATH	ABA	CYP707A2
AT2G29090.1	c88313_g2_i1	−1.37	−2.58	#N/A	ABAH1_ARATH	ABA	CYP707A2
AT1G52400.1	c80508_g1_i1	#N/A	1.46	#N/A	BGL34_ORYSJ	ABA	BGL1, ATBG1, BGLU18
AT1G52340.1	c84480_g1_i1	−1.83	−1.14	−1.86	C7A29_PANGI	ABA	SRE1, ISI4, ABA2
AT3G19270.1	c80869_g4_i2	#N/A	−1.95	−1.13	C7A52_PANGI	ABA	CYP707A4
AT3G19270.1	c83078_g3_i3	#N/A	−2.19	#N/A	C7A52_PANGI	ABA	CYP707A4
AT5G45340.1	c88211_g1_i2	#N/A	−1.61	−1.85	C7A52_PANGI	ABA	CYP707A3
AT1G30100.1	c99178_g3_i1	#N/A	−1.11	−0.77	NCED1_PHAVU	ABA	NCED5, ATNCED5
AT1G52340.1	c84480_g1_i2	#N/A	−1.71	−2.84	SILD_FORIN	ABA	SRE1, ISI4, ABA2
AT4G31500.1	c93443_g1_i2	#N/A	−1.54	−0.66	C71E1_SORBI	Auxin	CYP735A1
AT4G31500.1	c97022_g2_i1	#N/A	−1.72	#N/A	C71E1_SORBI	Auxin	CYP701A3, ATKO1, GA3
AT2G30770.1	c91695_g3_i1	#N/A	−1.65	−1.62	C78A7_ARATH	Auxin	CYP701A3, ATKO1, GA3
AT2G30770.1	c91695_g3_i3	#N/A	−1.74	#N/A	C78A7_ARATH	Auxin	CYP701A3, ATKO1, GA3
AT2G30770.1	c99171_g4_i6	−0.87	−1.09	#N/A	C78A7_ARATH	Auxin	CYP701A3, ATKO1, GA3
AT4G32540.1	c83588_g1_i1	1.26	1.27	#N/A	YUC_ARATH	Auxin	YUC, YUC1
AT1G17060.1	c93255_g4_i1	1.15	0.81	1.8	C7A29_PANGI	BR	CYP735A1
AT2G26710.1	c93255_g4_i5	#N/A	−1.56	#N/A	C7A29_PANGI	BR	CYP735A1
AT5G38450.1	c100283_g2_i2	0.67	−0.86	−0.94	C14A1_ARATH	Cytokinin	CYP735A1
AT2G36750.1	c88918_g1_i2	#N/A	−2.04	−2.09	SCGT_TOBAC	Cytokinin	UGT73C1
AT2G36800.1	c96045_g2_i1	#N/A	−1.74	#N/A	U91A1_ARATH	Cytokinin	UGT73C5, DOGT1
AT1G22400.1	c86370_g7_i2	1.71	1.58	#N/A	UGT2_GARJA	Cytokinin	UGT85A1, ATUGT85A1
AT1G22400.1	c98889_g4_i2	#N/A	−1.55	#N/A	UGT2_GARJA	Cytokinin	UGT85A1, ATUGT85A1
AT1G22400.1	c98889_g4_i4	#N/A	−3.08	−4.88	UGT2_GARJA	Cytokinin	UGT85A1, ATUGT85A1
AT1G15550.1	c89534_g1_i1	#N/A	−4.28	−1.52	ACCO1_ORYSI	GA	GA4, ATGA3OX1, GA3OX1
AT1G80340.1	c93875_g1_i4	#N/A	−1.06	−1.5	DIOX3_PAPSO	GA	ACO1, ATACO1
AT4G25420.1	c54669_g1_i1	#N/A	−5.34	#N/A	FL3H_PETCR	GA	ATGA20OX1, GA5, GA20OX1
AT4G25420.1	c81615_g1_i2	#N/A	−6.7	#N/A	FL3H_PETCR	GA	ACO1, ATACO1
AT4G25420.1	c94454_g1_i1	1.77	0.79	−0.65	FL3H_PETCR	GA	ACO1, ATACO1
AT4G02780.1	c97468_g5_i1	#N/A	−0.89	#N/A	NES2_FRAAN	GA	ATCPS1, CPS, CPS1, GA1
AT4G21200.1	c89719_g1_i1	−1.61	−2.04	−1.75	SILD_FORIN	GA	ACO1, ATACO1
AT4G21200.1	c89719_g1_i2	#N/A	−3.02	−1.21	SILD_FORIN	GA	ATACO2, ACO2
AT1G20510.1	c72977_g1_i1	#N/A	−2.01	−1.42	4CL2_SOYBN	JA	OPCL1
AT1G20510.1	c98993_g2_i2	#N/A	−0.96	#N/A	4CL2_SOYBN	JA	OPCL1
AT2G27690.1	c79501_g3_i1	#N/A	−2.16	#N/A	C86A2_ARATH	JA	CYP94C1
AT2G27690.1	c8721_g1_i1	#N/A	−2.06	−1.8	C86A2_ARATH	JA	CYP94C1
AT2G06050.2	c98433_g1_i3	#N/A	−0.14	0.43	OPR11_ORYSJ	JA	OPR3, DDE1, ATOPR3
AT1G22400.1	c90644_g3_i6	2.8	2.44	#N/A	KGLT_PETHY	SA	UGT85A1, ATUGT85A1
AT2G23620.1	c83239_g2_i1	#N/A	−1.53	#N/A	SABP2_TOBAC	SA	MES1, ATMES1

*Log*_*2*_*FC* Log_2_ fold change, *#N/A* no differential expression, *CPM* count per million reads, *FDR* false discovery rate

Among the three groups of flowering, hormone and sugar genes, many DETs (23) of hormone genes overlapped between non-transgenic and transgenic leaves of the NT:T plants suggesting that hormone genes might be involved in promoting flowering in the non-transgenic NT:T scions. Among these 23 shared DETs, there were four DETs representing two DE cytokinin genes similar to the CYP735A1 and UGT85A1 genes in *Arabidopsis*. CYP735A1 catalyzes biosynthesis of tZ-type cytokinins and increase in *CYP735A1* expression can enhance shoot growth in *A. thaliana*^54^. UGT85A1 is involved in *trans*-zeatin homeostasis and *trans*-zeatin responses and decreased expression of *UGT85A1* can lead to a low level of the *trans*-zeatin O-glucosides^55^. In *Arabidopsis*, cytokinins, auxin, and sugar are all involved in flower development and fertility^56^. In sweet cherry, a recent study has shown that the upregulated cytokinins played an inductive role in bud dormancy release^57^. Whether or not the two downregulated cytokinin genes were directly responsible for the reduced accumulation of c-ZR in the non-transgenic NT:T leaves remains to be resolved. Similarly, the functional roles of c-ZR in regulating plant flowering time control remains to be uncovered^58^.

Among the 23 DETs shared between transgenic and non-transgenic NT:T leaves, 4 were involved in the gibberellin biosynthesis pathway and they were annotated to be similar to two *Arabidopsis* genes, *GA2ox8* and *GA2ox1*. *GA2ox8* hydroxylates C_20_-GA precursors, and a repressed expression of *GA2ox8* promotes flowering^59–61^. Interestingly, *GA2ox8* was downregulated in both transgenic and non-transgenic leaves of the NT:T plants in this study, which is consistent with the expected expression change of the gene for promoting flowering in the scion. Also, in agreement is the *GA20ox1* which was upregulated in both transgenic and non-transgenic NT:T leaves. In *A. thaliana*, suppression of *GA20ox* in long days had little effect on flowering time, whereas in short days flowering was delayed^62^. Suppressed expression of *GA20ox1* would expect to lead to reduction of the synthesis of GA1 and then GA8^63^. Indeed, we observed the reduction of GA8 content in the non-transgenic NT:T leaves and the promotion of flower bud formation in the non-transgenic NT:T shoots.

ABA is known to suppress flowering^27^. Two DETs representing two ABA genes*, CYP707A2* and *ABA2*, were identified in both transgenic and non-transgenic NT:T leaves. Both were downregulated, which would expect to result in increasing ABA content^64^. Indeed, we observed in non-transgenic NT:T leaves, as described earlier, that the content of ABA, ABA-GE, 7′-Hydroxy-abscisic acid (7′OH-ABA), and *neo*-phaseic acid (*neo*-PA) were increased, but the content of DPA, PA, and *trans*-abscisic acid (t-ABA) were decreased, although not statistically significant.

Six DETs representing two auxin genes, *CYP71A13* and *YUC*, were among the 23 DETs shared by both transgenic and non-transgenic NT:T leaves. *CYP71A13* was downregulated while *YUC* was upregulated. *CYP71A13* catalyzes the conversion of indole-3-acetaldoxime to indole-3-acetonitrile (IAN) and its upregulation is expected to enhance the production of LAN and IAA^65^. On the other hand, downregulated *YUC1* represses the conversion of indole-3-pyruvic acid to IAA^66,67^. The expression behavior of YUC1 was consistent with the increased IAA content in the non-transgenic NT:T leaves.

A *CYP72C1* gene in the brassinosteroids biosynthesis pathway was upregulated in both transgenic and non-transgenic NT:T leaves. This gene, along with others, can inactivate brassinosteroids^68,69^ and consequently lead to delayed flowering in *A. thaliana*^70^. Unfortunately, the content of brassinosteroids was not assayed and therefore we could not make a connection between the expression of this gene and the changes of the brassinosteriods content in this study.

In the SA biosynthesis pathway, *UGT74F1* is involved in the conversion of SA or UDP-glucose to either SA-glucoside or SA glucose ester^71^ while *MES1* (MES1_ARATH) catalyzes the conversion of methyl salicylate (MeSA) to salicylic acid^72^. We observed increased expression of *UGT74F1* in both transgenic and non-transgenic leaves of the NT:T plants and reduced expression of *MES1* in the transgenic leaves, which agrees with the fact that the SA content in the non-transgenic NT:T leaves was much lower than that in the NT:NT leaves. Surprisingly, early flowering in the transgenic *VcFT*-OX plants was associated with decreased SA content, which was inconsistent with the previous reports in *A. thaliana* where increase of SA promotes flowering^43^.

Six DETs of three JA biosynthesis pathway genes (i.e., *CY94C1*, *OPR3*, and *OPCL1*) were detected and showed reduction of expression in the non-transgenic NT:T leaves. However, none of these DEGs showed differential expressions in the transgenic leaves. *CYP94C1* is known to convert 12-hydroxy-JA-Ile (12OH-JA-Ile) to the carboxy-derivative 12COOH-JA-Ile^73,74^ and therefore downregulation of *CYP94C1* could increase JA and JA-Ile synthesis by reducing their use as a source of precursors^73^. Indeed, we observed a decrease of the content of JA and JA-Ile in the non-transgenic NT:T leaves. *OPR3* and *OPCL1*, on the other hand, catalyze the formation of the JA precursors cyclopentane-1-octanoic acid (OPC-8:0) and OPDA-CoA and OPC-8:0-CoA, respectively^75,76^. Their downregulation would lead to an increase in methyl jasmonate synthesis, which was the case in the non-transgenic NT:T leaves.

There are 48 additional DETs of hormone genes which were found in the non-transgenic NT:T leaves, but not in the transgenic leaves (Table S4). Likewise, there were 75 DETs of hormone genes which were found in the transgenic leaves, but not in the non-transgenic NT:T leaves (Table S4). Not being able to detect the presence of these DETs in both tissues could be due to some biological reasons, but experimental errors and limited statistical power could also be a plausible explanation.

## Discussion

### Transgenic leaves are necessary for promoting early flowering

We previously demonstrated that constitutive expression of a *VcFT* in tobacco resulted in early flowering and plant dwarfing^46^. However, when the *VcFT*-expressing tobacco plants, with their leaves removed, were used as rootstocks, they did not significantly change flowering time of non-transgenic tobacco scions, clearly demonstrating that transgenic roots were not able to promote early flowering of the non-transgenic scions^77^. This observation is consistent with the results of NT:T grafting plants in cassava^23^, poplar^24^, apple^25^, and plum^26^ in which transgenic rootstocks with overexpression of *FT* or *FT* orthologues did not promote flowering in the recipient scions. In contrast, two NT:T woody shrubs, including blueberry in this study and *Jatropha* described in a previous report^22^, have shown that transgenic rootstocks with overexpression of *FT* or *FT* orthologues were able to promote flowering of NT scions. In both cases, transgenic leaves and branches were maintained when the plants were used as graft rootstocks, suggesting that these transgenic leaves are necessary for inducing flowering of the non-transgenic scions. This observation, consistent with the long-hold belief that leaves are the primary sources of florigen signals in inducing flowering^2^, provides a likely explanation for why previous NT:T grafting using transgenic *FT* rootstocks for promoting flowering of non-transgenic scions in woody species has often not been successful. Therefore, maintaining some leaves in a transgenic *FT* rootstock is necessary for successful induction of flowering in non-transgenic scions in a woody species.

### Suppressed *VcFT-*OX in roots

*FT* expression was scarcely detected in *Arabidopsis* roots due to its low level of accumulation^78,79^. In a previous study, we found that transgenic *VcFT* driven by a 35S promoter in transgenic blueberries was expressed at a much higher level in flower buds than in developing leaves and flowers^47^. In this study, we further observed that *VcFT-*OX transcripts in the transgenic young leaves (FPKM = 107.7) were about 1000-fold higher than in the transgenic roots (FPKM: 0.12) and 5000-fold higher than that in the leaves of non-transgenic blueberries. The lack of a significant increase of *VcFT* expression in the transgenic roots is very intriguing (transgenic roots FPKM: 0.12 vs. non-transgenic roots FPKM: 0.08), as a 35S promoter is well-known to be able to drive expression of a variety of genes in roots^80^. The possible explanations for this observation may include, but not be limited to, root-specific suppression of *VcFT* expression and/or rapid degradation of the transcribed *VcFT*-OX messages. Indeed, there are several potential layers of regulation existing in plants for regulation of *FT* expression, including those at transcriptional and posttranscriptional levels^81^.

### Florigenic signals in blueberries

So far, there is no solid evidence to support the hypothesis that FT/FT-like protein or mRNA is the florigen that undergoes long-distance transport through grafting unions from rootstocks to scions to promote flowering in woody plants. In this study, we observed 5000-fold higher expression of *VcFT* in the transgenic leaves than in the non-transgenic leaves. However, such a high level of *VcFT* expression in the transgenic leaves did not have an apparent impact on the accumulation level of *VcFT* in the non-transgenic leaves of NT:T plants. This observation confirmed the previous studies that *FT* RNAs do not likely function as the florigen which is transmitted to promote flowering. We did not measure *VcFT* proteins in this study and therefore do not have direct evidence that *VcFT* proteins were transmitted from the transgenic leaves to the non-transgenic leaves and buds of the NT:T plants for inducing flowering of the non-transgenic scions. However, the fact that many genes were differentially expressed in the non-transgenic leaves of NT:T plants and, most importantly, that non-transgenic scions on the NT:T blueberry were induced to flower clearly suggest that some florigenic signals, potentially including *VcFT* proteins, were transmitted.

The molecular process for how FT/FT-like proteins transmits from leaves to flower organs and regulate flowering pathway genes has been documented in annual species such as *Arabidopsis*^12^. A critical step in the process is that the transmitted FT proteins partner with FD proteins, interact with *SOC1*, *AP1*, *LFY* and others, and then trigger a cascade of changes of the expression of flowering pathway genes promoting flowering. In this study, we observed five such flowering DETs (based on the *Arabidopsis* flowering pathway genes) differentially expressed in both transgenic and non-transgenic leaves of the NT:T plants, including *SOC1* which is the direct target of *FT*. In addition to the flowering pathway genes, expression of 23 hormone pathway genes were also significantly affected by transgenic *VcFT*-OX in both transgenic leaves and non-transgenic NT:T leaves. Accompanying these hormone-related differentially expressed genes, the content of some corresponding phytohormones in the transgenic and non-transgenic NT:T leaves, compared with the non-transgenic NT:NT leaves, were also changed. This raises an interesting question about whether these hormones are part of the *FT*-based florigenic signals in promoting flowering.

Phytohormones affect plant flowering^1,3,33,82^ and have long been proposed as potential florigenic signals in promoting flowering^3^. However, such hormones have not been explicitly identified. Of the 41 phytohormone metabolites measured, 25 were detected in leaf tissues and some of them showed apparent content differences between transgenic NT:T leaves and non-transgenic NT:NT leaves. Interestingly, such differences for these metabolites were also observed between non-transgenic NT:T leaves and non-transgenic NT:NT leaves, although only GA8 and *cis*-zeatin riboside (c-ZR) showed statistically significant reduction in the non-transgenic NT:T leaves. However, the general trend is apparent that the non-transgenic NT:T leaves had intermediate levels of accumulation of these hormone metabolites between transgenic NT:T leaves and non-transgenic NT:NT leaves.

While we cannot make direct connections between the changed hormones content in the non-transgenic NT:T leaves and early flowering of buds on the non-transgenic NT:T scions, this study clearly demonstrated the involvement of hormones in the flowering promotion process. Whether the changed hormones content in the non-transgenic NT:T leaves resulted from direct migration of hormones from the transgenic NT:T leaves or from the activities of the hormone genes in the non-transgenic NT:T leaves regulated by potential transgenic *VcFT* proteins transported from the transgenic NT:T leaves cannot be determined in this study. Given that a generally low amount of FT protein is transported from leaves to buds in promoting flowering^12^ and there was an apparent gradient of hormone accumulation between the transgenic and non-transgenic NT:T leaves, we suspect that certain hormones from the transgenic leaves likely migrated into the non-transgenic NT:T leaves and then to buds to promote flowering of the non-transgenic NT:T scions in this study. This hypothesis may also explain why so many differentially expressed genes were identified in the differential expression analysis of the transgenic roots versus the non-transgenic roots, but no apparent increase of the *FT* RNAs was found in the transgenic roots. One likely explanation is that the hormones induced by transgenic *VcFT* in the transgenic leaves transmitted to roots and induced a cascade of changes of gene expression in the transgenic roots.

In this study, we have also observed some sugar synthesis pathway genes differentially expressed in transgenic leaves, transgenic roots, and non-transgenic NT:T leaves, indicating that sugar was likely involved in the *VcFT-*OX induced early flowering as well. Sugar is well known to affect plant flowering^83–87^. For example, in horticultural trees and bushes, sucrose modulates hormonal signaling in controlling bud outgrowth in *Rosa hybrida*^84^ and carbohydrates have been reported to serve as either a floral stimulus or an energy source in promoting flower bud formation^88^. The role of carbohydrates in regulating plant flowering is considered to be through flowering pathway genes (e.g., *FT* and *SOC1*) and phytohormones (e.g., GAs and JAs)^50,87^.

In conclusion, this study demonstrated that overexpression of *VcFT* in a transgenic blueberry promoted its early flowering and such transgenic blueberry, when used as a rootstock, promoted flowering of non-transgenic scions grafted to it. Previous failures for inducing flowering of non-transgenic scions by transgenic *FT* rootstocks in woody species are likely resulted from no accumulation of enough *FT* and/or *FT*-based flowering induction signals from the rootstocks whose leaves are usually not maintained. While *VcFT* proteins from the transgenic, overexpressed *VcFT* could still be the florigen in explaining early flowering of the grafted, non-transgenic scions in the present study, movement of the *VcFT* proteins from transgenic leaves to roots for explaining the extensive alteration of gene expression in the transgenic roots, in which transgenic *VcFT* expression was not enhanced, seems not likely on the basis of the known functions of the proteins. Unless proved otherwise, we suspect that the *VcFT*-induced changes of phytohormones in the transgenic leaves might be responsible, at least in part, for the observed molecular and phenotypic changes in both non-transgenic scions and transgenic roots of the NT:T plants.

## Materials and methods

### Transgrafting

Northern highbush blueberry cultivar ‘Aurora’ needs over 1000 chill units through winter dormancy for bud break. Non-transgenic ‘Aurora’ and transgenic ‘Aurora’ containing an overexpressed *VcFT* were used in blueberry transgrafting experiments. In our preliminary experiments conducted in 2016, transgrafting on five 5-year-old transgenic ‘Aurora’ plants of three transgenic events promoted flower bud formation in non-transgenic scions. In this study, all transgenic *VcFT*-OX-Aurora plants were derived from one representative transgenic event which was reported in our previous studies^45,47^. Non-transgenic ‘Aurora’ and transgenic *VcFT*-OX-Aurora plants were obtained from in vitro cultured shoots. The shoots were transplanted to growing media for rooting in July 2015 and the rooted shoots were grown through the winter in a heated greenhouse from December 2015 to September 2016. The 2-year-old plants received natural chilling treatment in a courtyard between greenhouses from September 2016 to May 2017. The 3-year-old plants were used for transgrafting experiments in May 2017 through cleft grafting. Transgrafting and self-grafting were attempted three times, to produce a total of 12 pairs of grafts involving *VcFT*-OX-Aurora (noted as T thereafter) and non-transgenic ‘Aurora’ (noted as NT thereafter) for each of the following combinations: self-grafted transgenic *VcFT*-OX-Aurora T:T, self-grafted non-transgenic ‘Aurosa’ NT:NT, and transgrafted NT scion on T rootstock NT:T (Fig. 1; Fig. S4). In this study, T:NT did not survive and therefore no data were collected from the grafting. For each pair of T and NT plants, the selected shoot tips of two branches (one for T and the other for NT) were reciprocally grafted using splice grafting. Self-grafting for T or NT plants was performed by cutting a shoot tip and rejoining the parts together. In each of these grafts, the leaves on the scions were removed and 5–10 leaves below the graft unions on the rootstocks were maintained. All grafted plants were grown for 3 weeks in a plant culture room at 25 °C under 16-h photoperiod of 35 μmol m^−2^ s^−1^ light from cool white florescent tubes. The surviving scions were photo-identified before the plants were moved to the greenhouse. In September of 2017, six plants each of NT:T, NT:NT, T and NT were moved to a growth chamber for chilling treatment for 2 months at 4 °C under 10-h photoperiod of 30 μmol m^−2^ s^−1^ light from cool white florescent tubes. Two each of NT:T and NT:NT grafted plants were grown in a greenhouse along with T and NT. Ten 6-year-old ‘Aurora’ plants grown in the courtyard were used as controls for monitoring ‘Aurora’ plant flowering. After receiving ~1440 CU in 2 months, the plants were moved to a greenhouse and grown at 22 °C under natural light conditions. Plant flowering was documented for all grafted plants as well as the controls from November 27, 2017 to May 23, 2018.

### Material for phytohormone and RNA-seq profiling

Three chilled NT:T and NT:NT plants, representing three biological replicates, were used for phytohormone analysis and RNA-seq sequencing. Young roots and leaves from each plant were harvested in January 2018 in NT:T and NT:NT plants. Young leaves, 1–2 g per plant, were harvested from multiple new shoots; half of these leaves were subjected to freeze-drying immediately and the other half were ground in liquid nitrogen and stored in a −80 °C freezer. From each NT:T plant, transgenic leaves (on the rootstocks) and non-transgenic leaves (on the scions) were harvested, respectively. From each NT:NT plant, leaves from scions were harvested. Young roots, 1–2 g, were excised from each plant, washed in double distilled water and blotted dry on filter paper. Similarly, for each root sample, half were subjected to freeze-drying, and the other half were ground in liquid nitrogen and stored at −80 °C.

### Phytohormone profiling

Freeze-dried tissues were used for profiling ABA and six ABA metabolites (ABAGE: Abscisic acid glucose ester, DPA: Dihydrophaseic acid, PA: Phaseic acid, 7′OH-ABA: 7′-Hydroxy-abscisic acid, neo-PA: *neo*-Phaseic acid, and *t*-ABA: *trans*-Abscisic acid), auxins [IAA: Indole-3-acetic acid, IAA-Asp: N-(Indole-3-yl-acetyl)-aspartic acid, IAA-Glu: N-(Indole-3-yl-acetyl)-glutamic acid, IAA-Ala: N-(Indole-3-yl-acetyl)-alanine, IAA-Leu: N-(Indole-3-yl-acetyl)-leucine, and IBA: Indole-3-butyric acid], cytokinins [*t*-ZOG: (t*rans*) Zeatin-O-glucoside, *c*-ZOG: (*cis*) Zeatin-O-glucoside, *t*-Z: (*trans*) Zeatin, *c*-Z: (*cis*) Zeatin; dhZ: Dihydrozeatin, *t*-ZR: (*trans*) Zeatin riboside, *c*-ZR: (*cis*) Zeatin riboside, dhZR: Dihydrozeatin riboside, iP: Isopentenyladenine, and iPR: Isopentenyladenosine], and 14 gibberellins (GA1, GA3, GA4, GA7, GA8, GA9, GA19, GA20, GA24, GA29, GA34, GA44, GA51, and GA53). These hormones were measured by the National Research Council of Canada, Saskatoon, SK S7N 0W9 (http://www.nrc-cnrc.gc.ca/eng/solutions/advisory/plant_hormone.html).

Fresh tissues of the same set of materials were used for RNA-seq analysis and quantification of the hormones SA, JA, and two JA metabolites (MeJA: Methyl jasmonate, JA-Ile: jasmonoyl isoleucine). The samples for hormone quantification were prepared following the protocols for *Arabidopsis*^89^ and were analyzed by the Mass Spectrometry and Metabolomics Core of Michigan State University. ANOVA and Tukey’s test were conducted using RStudio (Version 1.0.136).

### RNA-seq and differential expression analysis

Total RNA of each sample was isolated from ~200 mg of ground tissues using a CTAB method^90^, followed by using RNeasy Mini Kit for on-column DNase digestion and RNA purification (Qiagen, Valencia, CA, USA). The integrity of the RNA samples was assessed using the Agilent RNA 6000 Pico Kit (Agilent Technologies, Inc., Germany). All RNA samples submitted for RNA sequencing had an RNA quality score greater than 7.5 for roots and 8.0 for leaves. Sequencing (150-bp pair end reads) was conducted using the Illumina HiSeq4000 platform at the Research Technology Support Facility at Michigan State University (East Lansing, MI, USA). Thirty to sixty million reads were generated for each biological replicate. The FastQC program (www.bioinformatics.babraham.ac.uk/projects/fastqc/) was used to assess the quality of sequencing reads with the per base quality scores ranging from 30 to 40.

The RNA-seq reads of three biological replicates for each type of tissues were analyzed using Trinity^91^. The paired reads were aligned to the transcriptome reference Reftrinity^47^, and the abundance for each of a single read was estimated using the Trinity command “align_and_estimate_abundance.pl”. The Trinity command “run_DE_analysis.pl –method edgeR” was used to conduct differential expression analysis^91^. The differentially expressed (DE) genes or isoforms with the false discovery rate (FDR) value below 0.05 (*p-*value < 0.001) were used for further analyses of different pathway genes of blueberry. Fragments Per Kilobase of transcript per Million mapped reads (FPKM) were used to evaluate expression abundance. Most of the analyses were performed using the resources at the High Performance Computing Center at Michigan State University.

Pathway genes of nine phytohormones in *Arabidopsis*, including auxin, cytokinin, ABA, ethylene, gibberellin, brassinosteroid, jasmonic acid, salicylic acid, and strigolactones, were retrieved from RIKEN Plant Hormone Research Network and listed in Table S5. Similarly, pathway genes of sugar in *Arabidopsis* were identified and listed in Table S5. These *Arabidopsis* hormone and sugar genes were used as queries to blast against the transcriptome reference Reftrinity^47^ and the blueberry isoforms showing *E*-values less than −20 were identified and used for further analyses in various transcriptome comparisons. Blueberry flowering pathway genes identified in our previous study^47^ were used to analyze flowering-related DE isoforms identified in this study. Cytoscape 3.7.0 was used to construct gene networks of overrepresented gene ontology (GO) terms for the selected DETs under BiNGO’s default parameters with selected ontology file ‘GOSlim_Plants’ and selected organism ‘*A. thaliana*’^92,93^.

## Supplementary information

Figure S1 and Figure S2

Figure S3

Table S1

Table S2

Table S3

Table S4

Table S5

Table S6

Figure Table Legends
