# Supplementary material for: VcFT-induced mobile florigenic signals in transgenic and transgrafted blueberries
Source: Hortic Res. 2019 Sep 11;6:105. doi: 10.1038/s41438-019-0188-5 (PMC6804590; doi:10.1038/s41438-019-0188-5)
Supplement: Supplementary file 1 — Figure S1 and Figure S2 [file 41438_2019_188_MOESM1_ESM.docx]

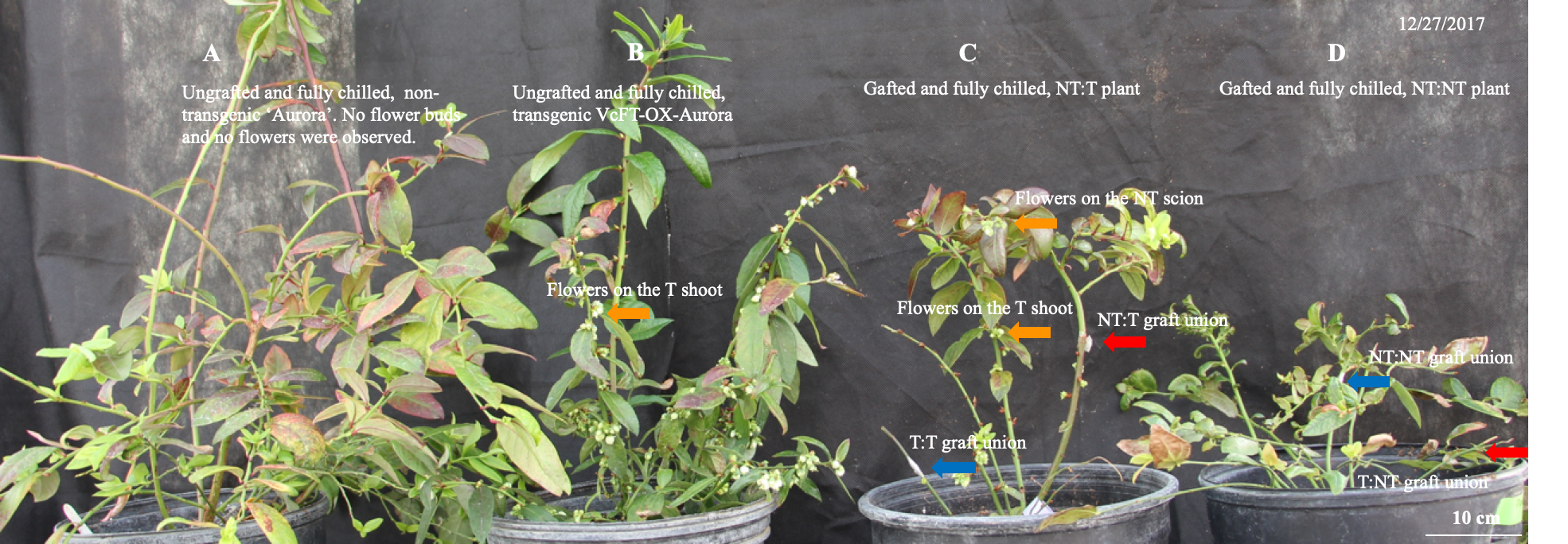


**Figure S1 Flower phenotypes of 3-year old, fully chilled blueberry plants.** (**A**) Ungrafted, non-transgenic ‘Aurora’ (NT) did not flower during the whole observation period from November 27, 2017 to May 23, 2018. (**B**) Ungrafted, transgenic VcFT-OX-Aurora (T) flowered. (**C**) Buds from both transgenic and non-transgenic shoots of the transgrafted NT:T plant flowered. (**D**) Self-grafted NT:NT (note: T:NT grafting failed) did not flower. Red arrow shows a NT:T graft union, blue arrows show T:T or NT:NT graft unions, and orange arrow shows flowers on a non-transgenic NT:T shoot.


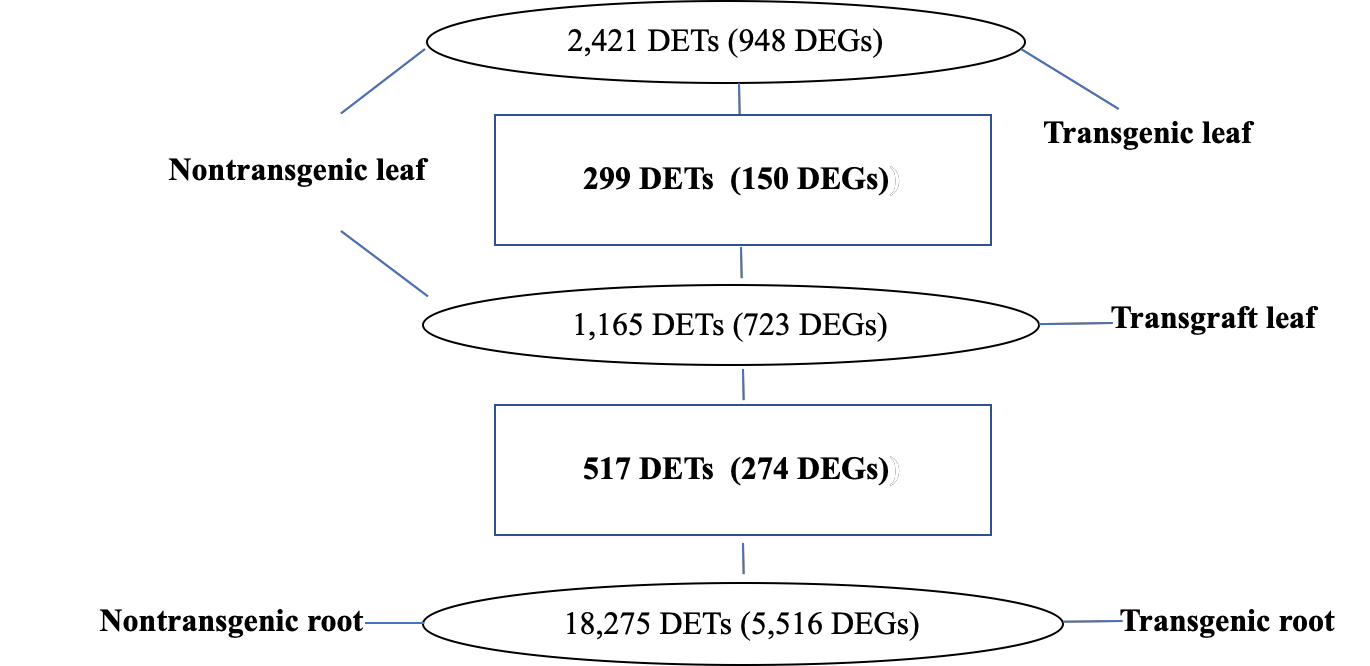


**Figure S2 Numbers of differentially expressed transcripts (DETs) or genes (DEGs) found between different comparisons of tissues at FDR < 0.05**. Ovals and associated lines show comparisons of various tissues and squares and associated lines indicate the number of overlapped DETs or DEGs between the two subjects involved.
