# Supplementary figures and images for: VcFT-induced mobile florigenic signals in transgenic and transgrafted blueberries
Source: Hortic Res. 2019 Sep 11;6:105. doi: 10.1038/s41438-019-0188-5 (PMC6804590; doi:10.1038/s41438-019-0188-5)

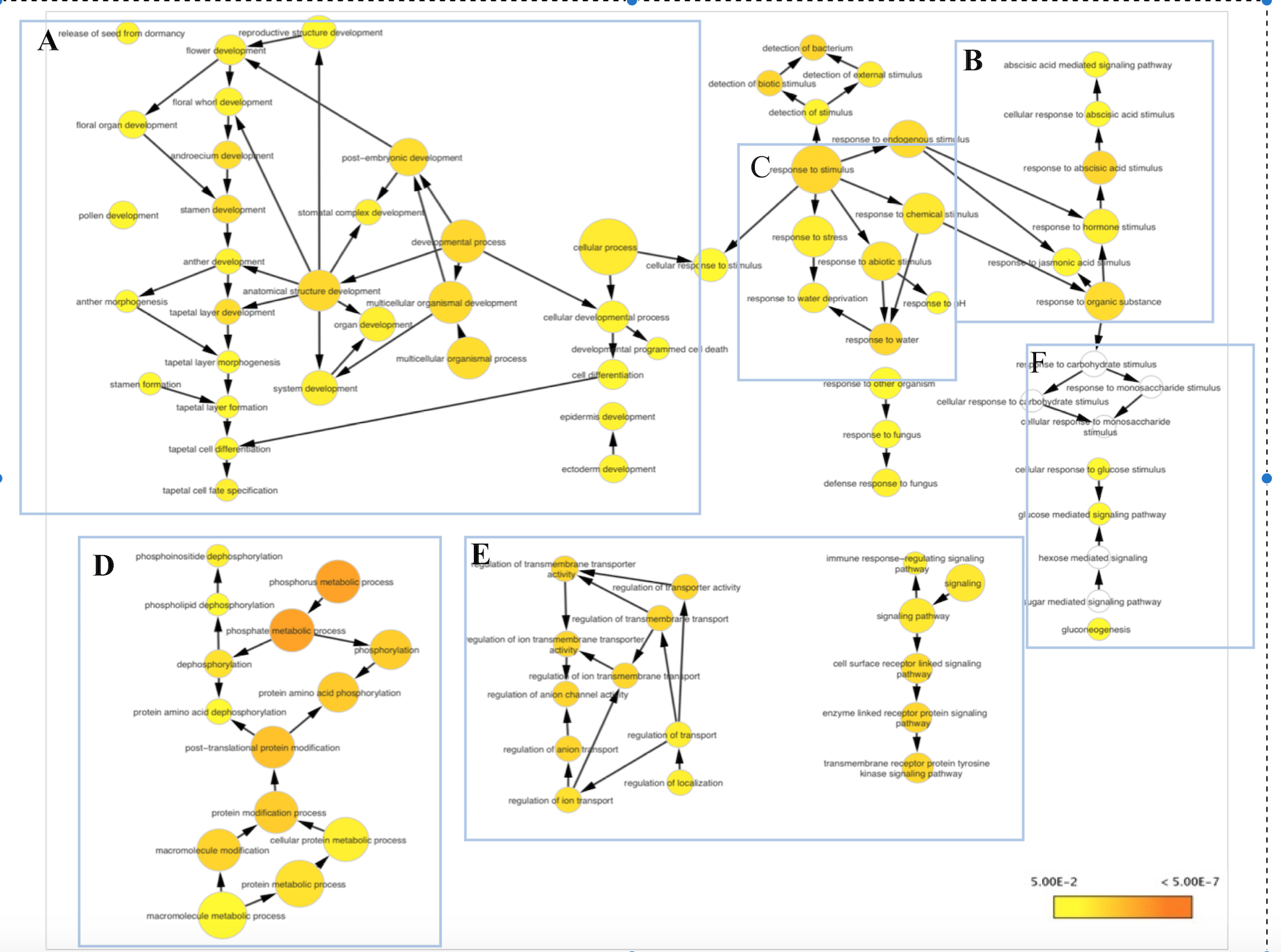

Supplement: Supplementary file 2 — Figure S3 [file 41438_2019_188_MOESM2_ESM.png]
