# Supplementary material for: VcFT-induced mobile florigenic signals in transgenic and transgrafted blueberries
Source: Hortic Res. 2019 Sep 11;6:105. doi: 10.1038/s41438-019-0188-5 (PMC6804590; doi:10.1038/s41438-019-0188-5)
Supplement: Supplementary file 9 — Figure Table Legends [file 41438_2019_188_MOESM9_ESM.docx]

**Figure S1** Flower phenotypes of 3-year old, fully chilled blueberry plants. (**A**) Ungrafted, non-transgenic ‘Aurora’ (NT) did not flower during the whole observation period from November 27, 2017 to May 23, 2018. (**B**) Ungrafted, transgenic VcFT-OX-Aurora (T) flowered. (**C**) Buds from both transgenic and non-transgenic shoots of the transgrafted NT:T plant flowered. (**D**) Self-grafted NT:NT (note: T:NT grafting failed) did not flower. Red arrow shows a NT:T graft union, blue arrows show T:T or NT:NT graft unions, and orange arrow shows flowers on a non-transgenic NT:T shoot.

**Figure S2** Numbers of differentially expressed transcripts (DETs) or genes (DEGs) found between different comparisons of tissues at FDR < 0.05. Ovals and associated lines show comparisons of various tissues and squares and associated lines indicate the number of overlapped DETs or DEGs between the two subjects involved.

**Figure S3** Gene networks of differentially expressed transcripts shared in the comparisons of non-transgenic NT:NT leaves versus non-transgenic and transgenic leaves from the NT:T grafts, respectively. (**A**) GO terms related to flower development. (**B**) Phytohormone-related GO terms. (**C**) Water-related GO terms. (**D**) Go terms related to phosphate metabolic process. (**E**) GO terms related to transporter and signaling pathway. (**F**) GO terms related to sucrose biosynthetic pathway. The gene ontology file of GOSlim_Plants in BiNGO was used to identify overrepresented GO terms (*P* < 0.05). Bubble size and color indicate the frequency of the GO term and the *P*-value, respectively.

**Table S1** Differentially expressed transcripts identified in the comparisons of transgenic NT:T leaves vs. non-transgenic NT:NT leaves, non-transgenic NT:T leaves vs. non-transgenic NT:NT leaves, and transgenic NT:T root vs. non-transgenic NT:NT roots. FDR (False discovery rate) < 0.05.

**Table S2** Differentially expressed transcripts shared between the two comparisons of transgenic NT:T leaves vs. non-transgenic NT/NT leaves and non-transgenic NT:T leaves vs. non-transgenic NT:NT leaves. FDR (False discovery rate) < 0.05.

**Table S3** Biological process Gene Ontology (GO) term analysis of differentially expressed transcripts shared in the comparisons of non-transgenic NT:NT leaves versus non-transgenic and transgenic leaves from the NT:T grafts, respectively.

**Table S4** Uniquely suppressed or enhanced DETs in the comparisons of transgenic NT:T leaves vs. non-transgenic NT:NT leaves, non-transgenic NT:T leaves vs. non-transgenic NT/NT leaves, and transgenic NT:T roots vs. non-transgenic NT:NT roots. FDR (False discovery rate) < 0.05.

**Table S5** Differentially expressed transcripts of flowering genes detected in the comparisons of transgenic NT:T leaves vs. non-transgenic NT:NT leaves, non-transgenic NT:T leaves vs. non-transgenic NT:NT leaves, and transgenic NT:T roots vs. non-transgenic NT:NT roots. FDR (False discovery rate) < 0.05.

**Table S6** Differentially expressed transcripts of sugar genes detected in the comparisons of transgenic NT:T leaves vs. non-transgenic NT:NT leaves, non-transgenic NT:T leaves vs. non-transgenic NT:NT leaves, and transgenic NT:T roots vs. non-transgenic NT:NT roots. FDR (False discovery rate) < 0.05.
